# Neoantigen-based cancer vaccines: a mechanistic and clinical review of personalised melanoma immunotherapy

**DOI:** 10.3389/fimmu.2026.1808146

**Published:** 2026-04-02

**Authors:** Xiyao Yu, Kai Fu

**Affiliations:** 1 School of Chemistry, Chemical Engineering and Biotechnology, Nanyang Technological University, Singapore, Singapore; 2 Department of Molecular, Cellular, and Developmental Biology, University of California, Los Angeles, Los Angeles, CA, United States

**Keywords:** clinical trials, cost-effectiveness, immune checkpoint inhibitors, immunotherapy, melanoma, neoantigen vaccine, patient perspective, personalised medicine

## Abstract

Melanoma, owing to its high tumour mutational burden (TMB) and inherent immunogenicity, has emerged as a prime target for neoantigen-based customised cancer vaccines. Such vaccines may synergise with immune checkpoint inhibitors (ICIs) by harnessing patient-specific mutations to trigger targeted T-cell responses. This review systematically summarises and evaluates the clinical evidence and molecular mechanisms underlying customised neoantigen vaccines in melanoma, based on key clinical trial data. A central finding is that vaccine platform choice strongly influences, rather than rigidly determines, the dominant immunological pathway. Messenger RNA (mRNA) platforms generally favour endogenous antigen expression and MHC class I presentation, often eliciting robust CD8^+^ cytotoxic T-cell responses. By contrast, synthetic long peptide (SLP) platforms are typically processed as exogenous antigens and frequently engage MHC class II presentation, thereby promoting substantial CD4^+^ T-helper responses. However, this distinction is not absolute, because exogenous peptides can also be cross-presented on MHC class I by professional antigen-presenting cells, enabling CD8^+^ T-cell priming under appropriate conditions. Clinical data reflects this, with the mRNA vaccine mRNA-4157 (KEYNOTE-942) demonstrating a significant recurrence-free survival (RFS) benefit in the adjuvant setting. This efficacy, however, is contingent on the “hot” tumour microenvironment (TME) of melanoma; “cold” tumours like glioblastoma (GBM) and ovarian cancer (OvCa) present TME-specific barriers (e.g., the Blood-Brain Barrier, immune exclusion) that demand distinct, combination-based vaccine strategies. This review deconstructs this heterogeneity and defines the primary bottlenecks to broad clinical adoption: (1) the need to bridge the “validation gap” by correlating AI prediction accuracy with clinical outcomes; (2) the formidable economic and logistical barriers, including a clinically vulnerable 8–16 week manufacturing wait that poses psychological and clinical risks to patients; and (3) navigating adaptive regulatory pathways for “n-of-1” therapeutics. The field awaits the pivotal Phase III clinical trial of V940-001 (NCT05933577), whose timeline has been extended to 2029. This reflects the logistical and biological complexities inherent in developing personalised vaccines, highlighting challenges in both manufacturing and subject recruitment. These remain key obstacles impeding the widespread clinical application of such vaccines.

## 1 Introduction

Melanoma, as an aggressive malignant tumour of melanocytes, exhibits one of the highest somatic tumour mutational burdens (TMB) among human cancers, primarily attributable to ultraviolet radiation-induced DNA damage. This high TMB results in a disproportionately high proportion of neoantigens—mutated peptides absent from the normal human proteome and possessing strict tumour specificity. The “foreign” nature of these neoantigens renders them ideal targets for T-cell-mediated immune recognition (1, 2).

The intrinsic immunogenicity of melanoma has been validated by the clinical success of immune checkpoint inhibitors (ICIs). Since 2011, immune checkpoint monoclonal antibodies targeting CTLA-4 (ipilimumab) and PD-1 (pembrolizumab, nivolumab) have revolutionised the prognosis for metastatic melanoma, enabling some to achieve durable long-term survival. However, a significant proportion of patients exhibit primary resistance (non-response) or develop acquired resistance following an initial response, mechanisms often involving immune evasion such as loss of T-cell infiltration or downregulation of antigen presentation pathways (3–6).

Personalised neoantigen vaccines, as tailored therapeutic strategies, hold promise in overcoming these limitations. By identifying patient-specific mutation profiles through next-generation sequencing and screening immunogenic epitopes via bioinformatics, vaccines can be engineered to precisely expand and diversify the patient’s endogenous anti-tumour T-cell repertoire. Unlike vaccines targeting tumour-associated antigens (TAAs)—which are typically overexpressed self-antigens within tumours—neoantigen-specific T cells are not constrained by central tolerance. Theoretically, they can induce high-affinity responses with minimal risk of off-target autoimmune reactions. Preclinical models and early trials demonstrate that such vaccines are safe and reliable, capable of inducing potent CD4^+^ and CD8^+^ T-cell responses. They may synergise with immune checkpoint inhibitors by activating novel T-cell clones that are “unleashed” following checkpoint blockade (7, 8).

While mid-stage randomized trials such as KEYNOTE-942 study of mRNA-4157 and the peptide-based trial NCT01970358, have shown encouraging signals of safety and efficacy, the protracted timeline of the pivotal V940–001 trial (now extended to 2029) underscores that translation to routine clinical practice remains a decade away, contingent on resolving manufacturing, economic, and regulatory bottlenecks. Significant variability in outcomes across different vaccine platforms (e.g., mRNA, peptide, dendritic cell) and methodological challenges in data synthesis have obscured a clear understanding of the true clinical potential. This review aims to critically synthesize the current clinical, mechanistic, economic, and regulatory landscape for personalised neoantigen vaccines in melanoma. It will deconstruct the observed heterogeneity by first establishing the core immunological principles differentiating the major vaccine platforms before performing a critical, corrected analysis of the clinical trial data (9–13).

## 2 The neoantigen immunotherapy pipeline: from silico to clinic

### 2.1 Genomic identification and in silico prediction

**Figure 1:**
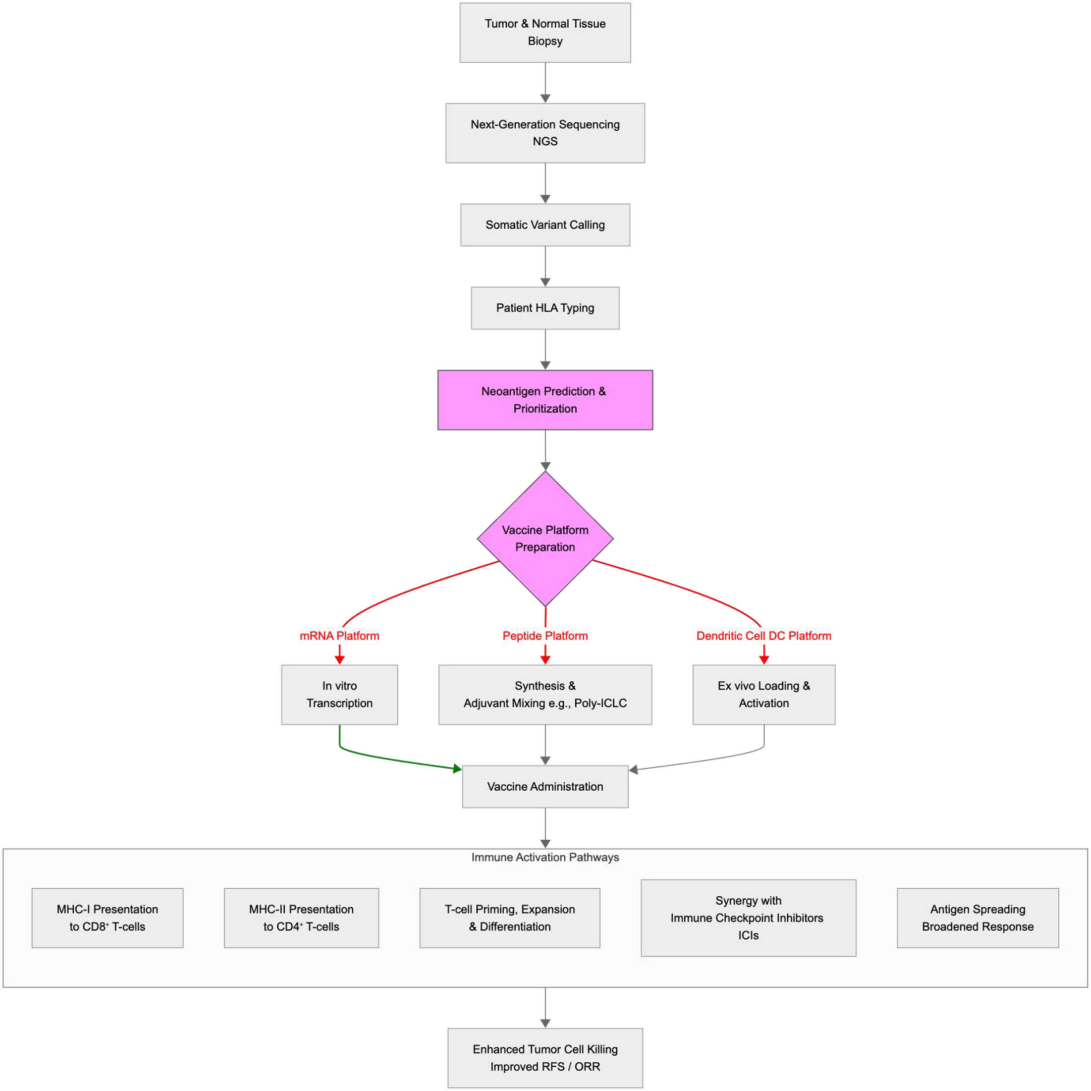
Neoantigen identification to immune activation process. This schematic illustrates the workflow: neoantigen identification (NGS, variant calling, HLA typing, epitope prediction) → vaccine preparation (platform-specific) → immune activation (TMB influence, MHC binding, T-cell activation pathways). Arrows depict sequential steps with key tools and challenges noted.

The generation of a personalised neoantigen vaccine is a complex, multi-step bioinformatic and manufacturing process (**Figure 1**). The workflow begins with the collection of tumour tissue and a matched normal sample (e.g., peripheral blood). Tumour and normal DNA are subjected to whole-exome sequencing (WES) or whole-genome sequencing (WGS) to identify somatic (tumour-specific) mutations. Simultaneously, tumour RNA sequencing (RNA-seq) is performed to confirm that these mutations are expressed and to quantify their transcript abundance (8, 13).

Following variant calling, the patient’s Human Leukocyte Antigen (HLA) haplotype is determined. Bioinformatic algorithms (e.g., NetMHCpan) are then used to predict which of the thousands of mutated peptides (neoepitopes) will bind with high affinity to the patient’s specific MHC Class I or Class II molecules. Rule-based pipelines that prioritize predicted HLA binding often exhibit low positive predictive value for true T-cell immunogenic neoantigens; benchmarking studies show that many top-ranked candidates fail downstream validation. They frequently fail to account for subsequent critical steps in antigen processing and presentation, such as proteasomal cleavage, peptide transport via TAP (Transporter associated with Antigen Processing), and the stability of the final peptide-MHC complex, leading to a high false-positive rate (12, 14).

### 2.2 The AI-driven prioritization challenge: accuracy vs. reproducibility

To overcome the limitations of simple binding-affinity algorithms, a new generation of Artificial Intelligence (AI) and machine learning models has been developed. These tools, such as imNEO, DeepNeoAG, and ImmuneMirror (**Table 1**), integrate multi-omic data, including mass spectrometry (MS)-verified immunopeptidomes, gene expression levels, and features of T-cell recognition. These models aim to provide a more composite prioritization score rather than a simple binding score (12, 15).

**Table 1:** Comparison of AI-based neoantigen prediction models.

Model	Input features	AUC	Experimental validation	Limitations
DeepNeoAG	Peptide sequences from melanoma antigens (no MHC allele info)	~ 0.90	5-fold cross-validation on CEDAR dataset; *in vitro* binding assays	Reproducibility issues in diverse HLA types; limited to melanoma sequences
ImmuneMirror	MHC binding affinity, stability rank, agretopicity, multi-omics (e.g., MS data)	0.87	Validated with hotspot mutations in ESCC/CRC/HCC; binding affinity assays with HLA-A02	Training data biases; poor transferability to non-hotspot mutations
imNEO	Epitope properties, antigen processing/presentation, T-cell interaction, tumour microenvironment, mutant-wildtype differential	>0.85	*In vivo* tumour growth inhibition models; antibody secretion tests; confirmed immunogenicity in multiple cancer datasets	Overfitting to specific cancer types; lacks independent clinical outcome validation

While these models report superior performance, with Areas Under the Curve (AUCs) often exceeding 0.85 in *in vitro* validation datasets (**Table 1**), a critical “validation gap” persists. The high AUCs reported by these models primarily reflect their accuracy at predicting *in vitro* peptide-MHC binding, an essential but insufficient proxy for *in vivo* T-cell activation and, more importantly, clinical efficacy. The translational link between a high prioritization score and a patient’s recurrence-free survival (RFS) has not yet been prospectively established (12).

Terminology used in this review: Binding refers to predicting peptide–MHC affinity/stability *in vitro*. Presentation refers to predicting whether a peptide is generated and displayed on the cell surface (processing, transport, and ligand elution), which is closer to *in vivo* biology but still not an immune response. Immunogenicity refers to demonstrable T-cell recognition/activation (functional assays and/or clinical immunomonitoring), and therefore should not be inferred from binding AUC alone.

An additional application of AI-guided prioritization that warrants explicit consideration is the selection of short peptides, or minimal epitopes, designed primarily for HLA class I presentation. In contrast to synthetic long peptides, which often require endosomal uptake and may preferentially expand CD4^+^ T-helper responses, short-peptide strategies can be used to enrich for candidate neoepitopes with high predicted HLA-I binding, favourable processing features, and greater likelihood of eliciting cytotoxic CD8^+^ T-cell responses. In this context, AI models are not merely ranking peptide–MHC affinity, but are increasingly being used to refine epitope length, anchor-residue suitability, presentation probability, and, in some cases, T-cell recognition features. These short-peptide approaches therefore represent a mechanistically distinct design strategy within the broader peptide-vaccine landscape, and their inclusion helps explain why peptide platforms should not be treated as uniformly CD4^+^-dominant (12, 14, 15). Nevertheless, short-peptide approaches are not universally superior, because their performance remains constrained by HLA restriction, peptide stability, and the risk of incomplete helper T-cell support.

This validation gap is exacerbated by what has been termed the “neoantigen algorithm reproducibility crisis”. Many AI models exhibit performance bias due to their training data. For example, models trained on datasets enriched for specific HLA alleles or on melanoma-specific sequences (which are abundant) may not generalize well to other cancer types or patients with different HLA haplotypes. This lack of standardization and poor transferability remains a major scientific and regulatory hurdle (12).

Although **Table 1** summarizes predictive performance, underlying architectural diversity contributes significantly to the observed spread in AUCs. DeepNeoAG utilizes a recurrent convolutional neural network trained on CEDAR peptide datasets, focusing on motif recognition independent of HLA allele context. ImmuneMirror instead applies ensemble gradient-boosting with integrated binding-stability and ligand-elution data, achieving higher biological interpretability. imNEO extends this framework by introducing TCR–epitope co-features, improving recall but at risk of melanoma-biased overfitting (12).

### 2.3 Addressing the reproducibility crisis in neoantigen AI models

While the field has made significant progress, two structural weaknesses underlie the reproducibility crisis in neoantigen prediction.

First, most machine learning pipelines are trained on heavily biased datasets—particularly overrepresented HLA-A02:01* alleles and melanoma-derived immunopeptidomes. As a result, model accuracy often collapses when applied to rarer HLA haplotypes or non-melanoma tumours. Second, benchmark datasets such as IEDB or CEDAR lack unified standards for peptide length, affinity thresholds, or negative-sample definition, inflating *in vitro* AUCs without reflecting true immunogenicity (12).

Emerging solutions emphasize cross-cohort benchmarking and synthetic augmentation of rare HLA alleles to enhance generalizability. In parallel, regulators have begun framing AI-based pipelines as “software as a medical device (SaMD)” components under Chemistry, Manufacturing and Controls (CMC) standards. This shift allows algorithmic validation to become a formal part of regulatory review, potentially transforming the current *ad hoc* research tools into auditable, clinical-grade systems (16–18).

## 3 Core immunological mechanisms: differentiating platform efficacy

The clinical heterogeneity observed in neoantigen vaccine trials is not random; it is, in large part, shaped by the distinct antigen-processing and presentation pathways preferentially engaged by different platforms. The choice of an mRNA- or peptide-based vaccine can bias the immune response toward particular T-cell compartments, but does not rigidly confine it to either CD8^+^ cytotoxic or CD4^+^ helper immunity (7, 8, 13).

### 3.1 The endogenous pathway: mRNA vaccines and CD8^+^ T-cell priming

Messenger RNA (mRNA) vaccines, typically encapsulated in lipid nanoparticles (LNPs), are delivered directly into the cytoplasm of cells, primarily antigen-presenting cells (APCs) such as dendritic cells. Once inside, the mRNA is translated by the host cell’s *own* ribosomes, producing the neoantigen protein *endogenously* (i.e., inside the cell) (8, 13).

This intracellular origin is immunologically critical. Endogenously synthesized proteins are processed by the proteasome into short peptides. These peptides are then transported by TAP into the endoplasmic reticulum, where they are loaded onto *MHC Class I* molecules. The peptide-MHC-I complex is then trafficked to the cell surface.

Presentation on MHC class I provides a major route for CD8^+^ cytotoxic T-lymphocyte (CTL) priming. This helps explain why mRNA vaccine trials often show strong CD8^+^-skewed responses, although accompanying CD4^+^ responses can also contribute meaningfully to anti-tumour immunity. This direct priming of CTLs—the immune system’s primary tumour-killing cells—provides a strong mechanistic rationale for the synergy observed between mRNA vaccines and anti-PD-1 ICIs. The vaccine primes an army of new tumour-specific killers, and the ICI releases the PD-1 “brake,” allowing them to execute their function (8, 9, 19).

### 3.2 The exogenous pathway: peptide vaccines, helper T-cell priming, and cross-presentation

In contrast, synthetic long peptide (SLP) vaccines, which are co-administered with an adjuvant (e.g., Poly-ICLC, Montanide) to stimulate APCs, are *exogenous* antigens. They are taken up from the extracellular space by professional APCs via endocytosis or phagocytosis (7, 13). It is also important to distinguish SLP vaccines from short-peptide or minimal-epitope formulations, which are often intentionally designed for MHC class I loading and CD8^+^ T-cell activation and therefore should not be mechanistically collapsed into the same category.

These exogenous peptides traffic through the endolysosomal pathway, where they are processed and loaded onto *MHC Class II* molecules. The peptide-MHC-II complex is then presented on the APC surface.

Presentation on MHC class II is a major mechanism through which SLP vaccines activate CD4^+^ T-helper cells, and this likely contributes to the high-frequency CD4^+^ responses reported in several peptide-vaccine trials. However, this pathway should not be interpreted as exclusive. After uptake by professional APCs, exogenous peptide antigens can also enter the MHC class I pathway through cross-presentation, thereby generating CD8^+^ T-cell responses under favourable biological and adjuvant conditions. Accordingly, peptide-based vaccines should be viewed as platforms that often favour CD4^+^ helper immunity but remain capable of inducing mixed CD4^+^/CD8^+^ responses, with the balance depending on peptide design, APC subset engagement, adjuvant choice, and antigen-processing efficiency. This mechanistic difference may underlie the more variable efficacy signals observed with peptide-based platforms (7, 13, 20).

Although the majority of current pipelines emphasize MHC-I–restricted CD8^+^ T-cell epitopes, the contribution of CD4^+^ T-cell responses via MHC-II presentation remains underexplored.

Prediction algorithms such as NetMHCIIpan 4.1 now allow high-throughput identification of HLA class II–restricted epitopes, though their accuracy remains lower than class I counterparts (14).

Incorporating MHC-II predictions may help explain why several peptide vaccine trials demonstrated robust CD4^+^ responses without corresponding clinical benefit, suggesting a need for balanced epitope selection (21, 22).

### 3.3 Vaccine platforms: a mechanistic and logistical comparison

The choice of platform involves a trade-off between the desired immune response, manufacturing speed, cost, and logistical stability. mRNA vaccines, for example, offer rapid manufacturing but require a stringent cold chain, whereas peptides are more stable but have a longer synthesis time. These differences are summarized in **Table 2** (13, 16).

**Table 2:** Mechanistic and logistical comparison of vaccine platforms.

Platform	Key immune pathway	Antigen processing	Dominant/typical T-cell response	Preparation time	Advantages	Limitations
mRNA	Predominantly MHC Class I, with secondary MHC II engagement	Endogenous (cytosolic)	Often CD8^+^-skewed, with supportive CD4^+^ responses	4–6 weeks	Strong cellular immunogenicity; rapid, scalable manufacturing	High cost; requires cold chain
Peptide/SLP	Predominantly MHC Class II, but may access MHC I via cross-presentation	Exogenous (endolysosomal; cross-presentation possible)	Frequently CD4^+^-dominant, but mixed CD4^+^/CD8^+^ responses are possible	6–10 weeks	Stable; simple production; flexible epitope design	CD8^+^ priming may be variable and depends on cross-presentation efficiency
Dendritic Cell (DC)	MHC Class I & II	Ex vivo loading	Dual (CD4^+^/CD8^+^)	8–12 weeks	Precise antigen loading; potent dual activation	Difficult to scale; high cost; complex ex vivo manufacturing

## 4 A critical review of clinical evidence in melanoma

### 4.1 Methodological note on synthesis: a narrative review, not a meta-analysis

A quantitative meta-analysis of neoantigen vaccine trials is precluded by the profound heterogeneity across studies. Trials differ in vaccine platform (mRNA vs. peptide), adjuvant used (e.g., Poly-ICLC, Montanide, CDX-1140), patient population (adjuvant stage III or IV vs. metastatic), comparator (ICI monotherapy vs. single-arm), and primary endpoints (RFS vs. ORR) (9, 11, 21–23).

Presenting these data in a single “forest plot” or calculating a “weighted average” effect size, as has been attempted, is statistically invalid and highly misleading. Combining heterogeneous endpoints like Hazard Ratios (HRs), which are time-to-event measures, and Objective Response Rates (ORRs), which are dichotomous proportions, on a single visual axis is methodologically unsound. Furthermore, attempting to “approximate” an HR from a Kaplan-Meier proportion ignores censored data and violates the proportional hazards assumption, rendering the estimate uninterpretable. Any weighting by simple sample size ( n), rather than by the inverse variance of the effect estimate, is not a valid meta-analytic technique.

Therefore, this review will not conduct a meta-analysis. Instead, it adheres to the Synthesis Without Meta-analysis (SWiM) guidelines by presenting a critical narrative synthesis, with results from the six key trials summarized descriptively in **Table 3**. A risk-of-bias assessment (**Table 4**) highlights that all single-arm trials included suffer from a high risk of selection and reporting bias (24).

**Table 3:** Summary of key clinical trials of personalised neoantigen vaccines in melanoma.

Trial ID	Platform/agent(s)	n	Population	Key efficacy and immunogenicity outcomes	Status/limitations
KEYNOTE-942 (NCT03897881)	mRNA (mRNA-4157) + Pembrolizumab	157	Adjuvant (Resected Stage IIIB-IV)	Efficacy (3-yr): RFS HR 0.510 (95% CI 0.288–0.906); DMFS HR 0.384. Sustained CD4^+^/CD8^+^ responses.	Positive. Phase 2b, Randomized.
NCT01970358	Peptide (NeoVax) + Poly-ICLC	8 (LTFU)	Adjuvant (Resected Stage IIIB-IV)	Efficacy (~4-yr): 6 of 8 (75%) patients disease-free. Immunog: Persistent, diversified memory T-cell responses.	Positive. Phase 1, Single-arm.
NCT05309421	Peptide (EVX-01) + Pembrolizumab	16	Metastatic (Unresectable)	Efficacy (2-yr): 75% ORR (12/16) (95% CI 0.51–0.90). Durable: 92% (11/12) of responses sustained at 24 mos.	Positive. Phase 2, Single-arm.
NCT03929029	Peptide (NeoVax) + Montanide + Ipi/Nivo	11	Metastatic	Efficacy (Final): 36% ORR (4/11) (95% CI 0.15–0.65). Immunog: Responses in 8/11.	Inconclusive. Phase 1b. Final results posted Oct 2024.
NCT04072900	Peptide + Toripalimab (Anti-PD-1)	30	Metastatic	Efficacy: 10% ORR (3/30) (95% CI 0.03–0.26).	Negative. Phase 1, Single-arm.
NCT04364230	Peptide (Shared Ag + neoAg-mBRAF) + Adjuvants	22	Adjuvant (Resected, Disease-Free)	Immunog: 27% (6/22) *ex vivo* CD4^+^ response to 6MHP (shared Ag). No *ex vivo* response to neoantigen (1 patient IVS-positive).	Negative (Immunog.). Phase 1/2. No efficacy endpoint; 73% (16/22) was adjuvant dose, not RFS.

**Table 4:** Risk of bias assessment (RoB 2 for randomized trials; ROBINS-I domains for non-randomized single-arm studies).

Domain	KEYNOTE-942	NCT01970358	NCT03929029	NCT04364230	NCT04072900	NCT05309421	Overall assessment
Selection Bias	Low (Randomized)	High (Single-arm)	High (Single-arm)	High (Single-arm)	High (Single-arm)	High (Single-arm)	High
Performance Bias	Moderate (Partial blinding)	Moderate (Partial blinding)	Moderate (Partial blinding)	Moderate (Partial blinding)	Moderate (Partial blinding)	Moderate (Partial blinding)	Moderate
Detection Bias	Low (Objective endpoints)	Low (Objective endpoints)	Low (Objective endpoints)	Low (Objective endpoints)	Low (Objective endpoints)	Low (Objective endpoints)	Low
Attrition Bias	Low (Low dropout)	Low (Low dropout)	Low (Low dropout)	Low (Low dropout)	Low (Low dropout)	Low (Low dropout)	Low
Reporting Bias	Moderate (Selective outcomes)	High (Selective outcomes)	High (Selective outcomes)	High (Selective outcomes)	High (Selective outcomes)	Moderate (Selective outcomes)	High
Other (Heterogeneity)	High (Patient variability)	High (Patient variability)	High (Patient variability)	High (Patient variability)	High (Patient variability)	High (Patient variability)	High

Bias assessment followed qualitative domains adapted from the ROBINS-I framework, including selection bias, confounding, and measurement bias. Trials with non-randomized design were rated high in at least one domain but varied in magnitude. A semi-quantitative bias score was intentionally omitted in accordance with SWiM narrative-synthesis guidance; qualitative grading was used to reflect methodological robustness without introducing pseudo-numeric precision.

### 4.2 Summary of clinical trial evidence

The clinical evidence for neoantigen vaccines in melanoma is derived from a small number of key Phase I/II trials (**Table 3**). A critical re-examination of the data from these trials, including recent 2024–2025 updates, reveals a more complex and nuanced picture than previously reported (9, 11, 21–23, 25, 28).

### 4.3 Visualizing platform-specific clinical outcomes

**Figure 2:**
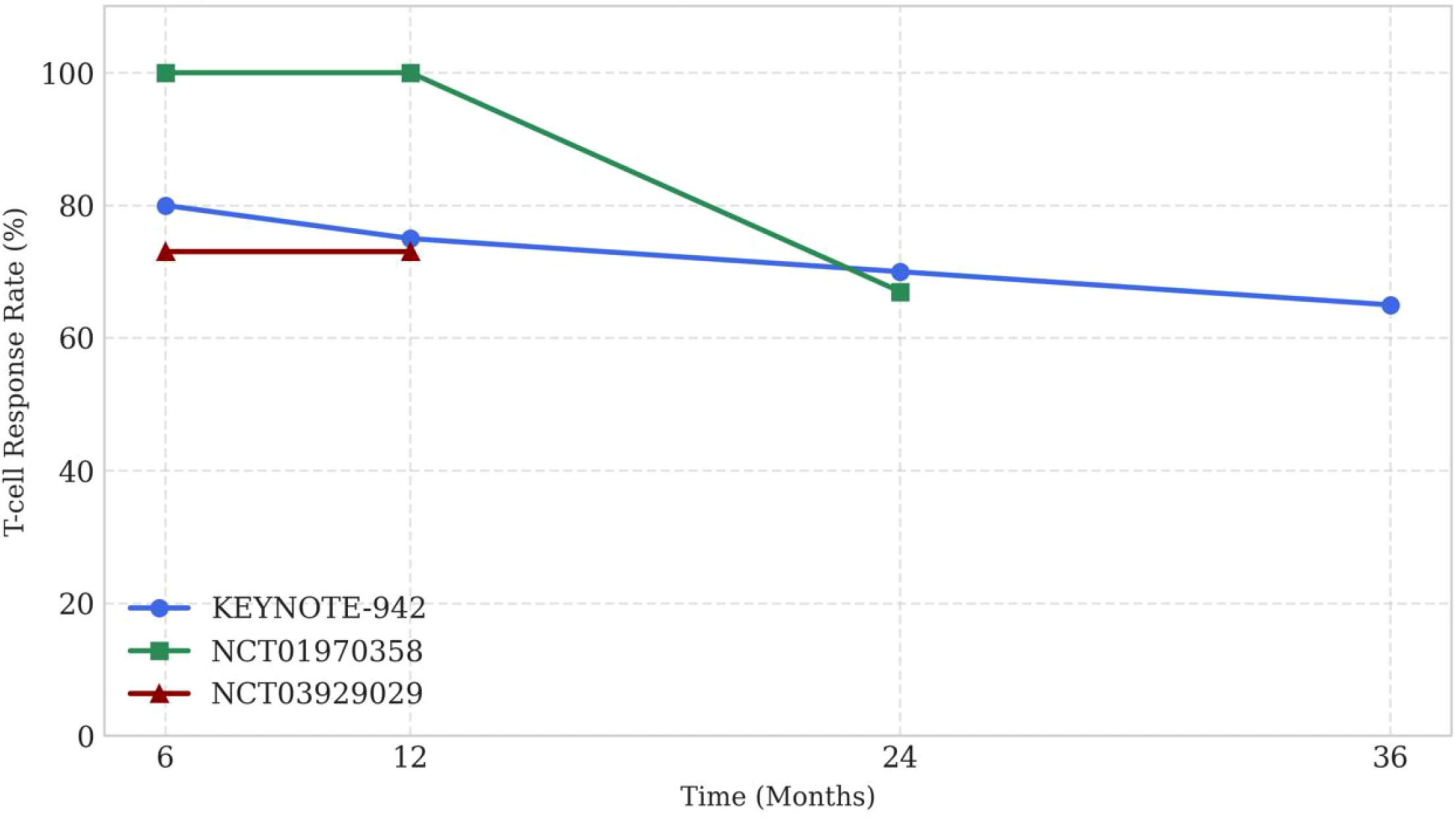
Durability of T-cell responses in selected neoantigen vaccine trials. X-axis: Time (Months); Y-axis: T-cell Response Rate (%). Data for KEYNOTE-942: ~80% at 6 months, 75% at 12, 70% at 24, ~65% at 36 (preliminary data from conference abstract; final results may update); NCT01970358: 100% at 6/12, 67% at 24; NCT03929029: 73% at 6/12; others limited. **(B)**. Platform-specific clinical outcomes by AI sophistication. Each data point represents one clinical trial: the x-axis denotes vaccine platform (mRNA, peptide, or dendritic cell), the y-axis represents the principal endpoint (RFS or ORR), and bubble size corresponds to sample size. Colour intensity reflects the level of AI integration—ranging from rule-based affinity predictors (light) to deep-learning platforms (dark).

To clarify the relationship between vaccine type, prediction pipeline, and outcome, **Figure 2B** stratifies the key melanoma trials by platform and AI model sophistication.

Although the clonal architecture of tumours has been recognized as a major determinant of vaccine efficacy, only a minority of ongoing trials explicitly incorporate clonal analysis in antigen selection. Although tumour clonality is increasingly recognized as important, explicit clonality-informed antigen selection is not consistently reported across trials, and thresholds/pipelines remain non-standardized.

In practice, clonality-informed antigen selection depends not only on mutation detection by NGS, but also on how variant allele fraction is interpreted after accounting for tumour purity, local copy-number status, sequencing depth, and sampling bias. As a result, there is no universally accepted NGS-based cutoff that cleanly separates clonal from subclonal neoantigens across studies. For this reason, clonality should be treated as a probabilistic prioritization feature rather than a binary rule and ideally interpreted together with RNA expression and antigen-presentation likelihood.

Incorporating clonal frequency estimates into antigen prioritization pipelines may improve the likelihood of targeting truncal neoantigens that persist across metastases. However, real-time implementation remains constrained by the computational cost and lack of standardized thresholds for clonality calls (26, 27).

Trials employing deep-learning–guided neoantigen selection (e.g., EVX-01, imNEO) cluster in the upper-right quadrant, reflecting higher ORR and durability, even with comparable baseline TMB and disease stage (**Supplementary Table 1**) (28).

### 4.4 Synthesis of clinical signals

The corrected trial data (**Table 3**) reveals distinct patterns.

For mRNA Platform (KEYNOTE-942), the randomized Phase 2b KEYNOTE-942 trial provides the highest-quality evidence to date. The combination of the mRNA vaccine mRNA-4157 with pembrolizumab resulted in a clinically and statistically significant improvement in RFS. The 3-year update confirmed a durable benefit, with an RFS HR of 0.510 (49% risk reduction) and a distant metastasis-free survival (DMFS) HR of 0.384. In the randomized phase 2b KEYNOTE-942 trial, the observed 49% relative reduction in recurrence or death was demonstrated within a cohort of completely resected high-risk stage IIIB-IV melanoma. Because recurrence risk differs across TNM categories, direct comparison with external populations should be interpreted cautiously unless stage-matched subgroup data are available. This result strongly supports the hypothesis that vaccine-induced cellular immunity, including a substantial cytotoxic T-cell component, can add tangible benefit to checkpoint blockade (9, 19, 25).

For Peptide Platforms (A Heterogeneous Picture), they show significant variability.

#### 4.4.1 Positive signals

NCT01970358, the first-in-human NeoVax peptide study, demonstrated remarkable long-term persistence of T-cell memory and durable disease control, with 75% of patients (6/8) remaining disease-free at a median follow-up of nearly 4 years. More recently, NCT05309421 (EVX-01) showed a high 75% ORR in *metastatic* patients, and critically, these responses were highly durable, with 92% (11/12) sustained at 2 years. This trial’s success was explicitly linked to its AI-driven prediction platform, which reportedly achieved 81% accuracy in predicting T-cell responses (7, 11, 20, 28).

#### 4.4.2 Negative/inconclusive signals

The narrative is balanced by clear negative signals. NCT04072900, which combined a peptide vaccine with the anti-PD-1 inhibitor toripalimab in metastatic patients, reported a dismal 10% ORR (3/30) (21).

#### 4.4.3 The NCT04364230 correction

A critical re-analysis of NCT04364230 is required. This trial was previously misinterpreted as showing a 73% (16/22) relapse-free rate. However, recent 2024 data (SITC poster 1466) clarifies this is factually incorrect. The 22 patients were enrolled *disease-free*. The “73%” figure (16/22) referred to the proportion of patients who received the maximum dose of the *adjuvant*, not an efficacy outcome. The actual primary endpoints were safety and immunogenicity, and the latter was poor: a CD4^+^ T-cell response to the shared antigen component was seen in only 27% (6/22) of patients *ex vivo*, and responses to the neoantigen component (neoAg-mBRAF) were essentially undetectable *ex vivo* (22).

This synthesis suggests that vaccine success is not a simple “mRNA vs. peptide” dichotomy. Rather, it is contingent on the *quality* of the antigen prediction (e.g., the 81% accuracy in the successful EVX-01 trial) and the biological context of the patient (**Figure 5**). As summarized in **Supplementary Table 1** and visualized in **Figure 2B**, trials using deep-learning–guided neoantigen selection appear to cluster with higher response rates and durability, supporting the view that prediction quality may be a dominant driver of clinical benefit (26, 28).

### 4.5 Emerging biomarkers for neoantigen vaccine response

While neoantigen-based vaccines have demonstrated promising efficacy in select melanoma cohorts, predictive biomarkers that delineate responders from non-responders remain underdefined. A multidimensional biomarker strategy is therefore essential to guide patient selection (**Table 5**), monitor vaccine-induced immunity, and optimize clinical outcomes.

**Table 5:** Emerging biomarkers for neoantigen vaccine response.

Biomarker	Type	Evidence of predictive value	Key limitations
Tumour Mutational Burden (TMB)	Predictive	Correlates with neoantigen load and ICI response	Not all mutations generate immunogenic peptides
Neoantigen Clonality	Predictive	Truncal antigens linked to durable T-cell responses	Requires deep sequencing; difficult to quantify subclonal targets
IFN-γ Signature/MHC-I Expression	Predictive	Reflects immune readiness and antigen presentation	Influenced by inflammation and therapy-induced modulation
ctDNA Clearance	Dynamic	Tracks vaccine-induced tumour regression	Low sensitivity in minimal disease states
TCR Repertoire Diversity	Dynamic	Indicates clonal expansion and persistence	Requires paired pre/post samples
Composite AI Models	Integrative	Multimodal predictor of clinical benefit	Lack of standardization and external validation

a. Predictive biomarkers

Tumour mutational burden (TMB) has been consistently correlated with the abundance of neoantigens; however, its predictive precision is limited by intertumoural heterogeneity and non-immunogenic passenger mutations. Clonality of neoantigens—particularly those derived from truncal mutations—has shown stronger association with durable responses compared to subclonal targets. In parallel, baseline interferon-γ (IFN-γ) signalling and MHC-I expression levels serve as key immunocompetence markers, influencing both antigen presentation and cytotoxic T-cell priming efficiency (2, 26, 29–31).

b. Dynamic monitoring biomarkers

Dynamic immune readouts have become critical in capturing real-time therapeutic efficacy. Circulating tumour DNA (ctDNA) dynamics and longitudinal T-cell receptor (TCR) repertoire tracking are being evaluated as potential response markers in neoantigen-vaccine studies. These approaches enable quantification of clonal expansion and persistence of vaccine-primed T cells, offering a more granular readout of immune kinetics than static assays (12, 13).

c. Integrative modelling and composite scores

Recent efforts have focused on integrating genomic, transcriptomic, and immunophenotypic metrics into composite predictive models. Frameworks based on AI-driven ensemble predictors combine TMB, neoantigen clonality, and immune signatures to stratify patients into high- and low-likelihood responders. Such composite indices could evolve into standardized metrics for clinical trial stratification and regulatory validation (12).

In summary, predictive biomarkers must evolve beyond TMB-centric paradigms toward dynamic, integrative models capturing both tumour-intrinsic and immune-contextual variables.

## 5 Determinants of efficacy and mechanisms of resistance

### 5.1 The clonality-clonality divide: targeting truncal vs. subclonal neoantigens

A primary mechanism of vaccine failure and tumour immune escape is intra-tumour heterogeneity (ITH). A tumour is not a monolith but an evolving ecosystem of distinct subclones. Mutations can be “clonal” (truncal), meaning they arose early and are present in all tumour cells, or “subclonal” (branched), existing in only a subset of cells (27).

Although clonality is already recognized as important, its impact cannot be overstated. A landmark meta-analysis of 12 checkpoint inhibition trials provided a definitive insight: high *subclonal* TMB confers *no clinical benefit* to CPI therapy, whereas high *clonal* TMB is *significantly correlated with better overall survival* (p = 2.9×10^−7^) (26).

The implication for vaccine design is profound. A vaccine targeting a subclonal neoantigen may successfully eliminate the subclones that express it, but this merely “prunes the tree” and selects for the growth of other subclones that lack the target antigen, leading directly to relapse. Therefore, truncal or highly clonal neoantigens are generally attractive vaccine targets, although effective vaccine design must also account for subclonal architecture, immunoediting, and dynamic antigen loss. The failure to prioritize clonal neoantigens, a bioinformatic challenge, is a likely explanation for the modest efficacy seen in trials like NCT04072900 (21, 26, 27).

### 5.2 The tumour microenvironment as a barrier: lessons from non-melanoma trials

The highly immunogenic, “hot” TME of melanoma is permissive for immunotherapy. Trials in “colder,” more immunosuppressive tumours, such as glioblastoma (GBM) and ovarian cancer (OvCa), provide crucial insights into failure mechanisms. However, the analysis in the original draft contained critical misinterpretations of key studies (32–34).

#### 5.2.1 Glioblastoma and the dexamethasone confounder

The draft cited the Keskin et al. *Nature* 2019 trial as a failure due to general immunosuppression. The reality is more specific and iatrogenic. This Phase Ib trial (NCT03422094) of a peptide neoantigen vaccine did generate T-cell responses that were shown to migrate into the intracranial tumour. The critical finding, however, was that this response was completely dependent on concomitant medication: patients treated with dexamethasone—a potent steroid routinely given to GBM patients to reduce cerebral edema—showed no vaccine-induced T-cell response. In contrast, the few patients who did not receive dexamethasone showed a strong response. This demonstrates that a standard-of-care medication can completely ablate vaccine efficacy (32, 35).

#### 5.2.2 Ovarian cancer and the low-TMB myth

The draft cited the Bobisse et al. *Nat Commun* 2018 study as a failed vaccine trial demonstrating immune escape. This is a severe misreading of the evidence. The Bobisse study was *not* a vaccine trial. It was a foundational immunopeptidomic study of immunotherapy-naive OvCa patients. Its finding was, in fact, highly optimistic: it demonstrated for the first time the “sensitive and frequent identification” of high-avidity, neoepitope-specific CD8^+^ T-cells in TILs, even in a “cold” tumour with a low TMB. The Bobisse paper proves that the *targets exist*. The *actual* reason for vaccine failure in OvCa (e.g., in trials like NCT03199040) is the low *quantity* of neoantigens and the presence of a profoundly immunosuppressive TME that creates physical and chemical barriers to T-cell infiltration and function (33, 34, 36, 37).

### 5.3 A comparative analysis: the immunological divide in melanoma, GBM, and ovarian cancer

The success of neoantigen vaccines in melanoma, and their relative struggles elsewhere, is not arbitrary. It is a direct function of fundamental differences in tumour immunobiology. TMB provides the *targets*, but the TME dictates the *strategy*. A comparison of these three distinct cancers (**Table 6**) illuminates the landscape of challenges (26, 34).

**Table 6:** Comparative immunobiology and vaccine strategies (melanoma vs. GBM vs. OvCa).

Characteristic	Melanoma (SKCM)	Glioblastoma (GBM)	Ovarian cancer (OvCa)
Median TMB (Muts/Mb)	High (relative to many solid tumours)	Low (Median ~5.9)	Low (Median ~3.6–5.0)
TME Phenotype	“Hot”/Inflamed (High T-cell infiltration)	“Cold”/Immune-Privileged (Low T-cell infiltration)	“Cold”/Immune-Excluded (T-cells blocked from entry)
Primary Immune Barrier	T-cell exhaustion (PD-1/PD-L1 expression)	Blood-Brain Barrier (BBB); profound TME immunosuppression	Physical T-cell exclusion (high IFP, stroma); high MDSC/Treg infiltration
Key Vaccine Strategy	Systemic mRNA or peptide vaccine + anti-PD-1 ICI (e.g., KEYNOTE-942)	Localized peptide vaccines; must avoid concomitant dexamethasone	Combination strategy (e.g., DC vaccine + chemo) to “heat up” TME

#### 5.3.1 Melanoma as the “hot” archetype

Melanoma is the “best-case scenario” for immunotherapy. It is an immunologically “hot,” or inflamed, tumour. Its relatively high TMB is driven by UV radiation and provides a rich source of neoantigens. Its TME is characterized by high T-cell infiltration. The primary immune barrier is T-cell exhaustion, driven by PD-1/PD-L1 expression. Therefore, the strategy is logical: a systemic vaccine (like mRNA-4157) primes new T-cells, and an ICI (like pembrolizumab) “releases the brakes” on the exhausted, pre-existing T-cells (1, 9, 29–31).

#### 5.3.2 Glioblastoma: the “cold,” immune-privileged barrier

GBM is an immunologically “cold” tumour. It has a very low TMB (median ~5.9 Muts/Mb), severely limiting the number of potential targets. Its TME is profoundly immunosuppressive. However, the dominant challenge is the Blood-Brain Barrier (BBB), a unique anatomical structure that physically shields the tumour from systemic T-cell infiltration. This unique location mandates a different strategy. Real-world studies have consequently focused on localized, personalised peptide vaccines. As noted previously, the strategy must also account for iatrogenic immune suppression from standard-of-care dexamethasone, which can single-handedly ablate vaccine efficacy (32, 35, 38).

#### 5.3.3 Ovarian cancer: the “cold,” immune-excluded barrier

OvCa is also classified as “cold”, with a low TMB (median ~3.6–5.0 Muts/Mb) offering few targets. Its TME is not just immunosuppressive but “immune-excluded”. It is characterized by a “paucity” of infiltrating CD8^+^ T-cells. This exclusion is driven by physical and chemical barriers, including high interstitial fluid pressure (IFP) and a dense stroma that prevent T-cells from entering the tumour bed, as well as a high influx of inhibitory cells like MDSCs and Tregs. A systemic vaccine monotherapy is futile, as the T-cells it generates cannot reach the tumour. Therefore, successful strategies in OvCa must first “heat up” the TME and break this exclusion, requiring combinations with chemotherapy, oncolytic viruses, or dendritic cell vaccines (33, 34, 36, 37).

### 5.4 Beyond clonality: molecular mechanisms of immune escape

Beyond subclonal diversity, multiple molecular mechanisms can undermine vaccine-induced immunity. Loss of antigen presentation—via β2-microglobulin (β2M) mutations, MHC-I heavy chain loss, or TAP1/2 transport defects—renders tumour cells invisible to cytotoxic T lymphocytes despite robust priming (6, 26).

Similarly, chronic antigen exposure may drive T-cell exhaustion characterized by PD-1^high^TIM-3^+^LAG-3^+^phenotypes, reducing effector function (6).

Under selective pressure, tumours may undergo immunoediting, progressively deleting target neoantigens or downregulating interferon-γ signalling pathways (6, 26).

Addressing these escape routes will require rational combination strategies—such as pairing vaccines with oncolytic viruses, STING agonists, or epigenetic modulators—to restore antigen presentation and reinvigorate exhausted T cells (13, 39).

## 6 Safety and tolerability profile across platforms

While efficacy signals of neoantigen-based vaccines are increasingly robust, systematic evaluation of safety outcomes remains limited. Across early-phase trials, treatment-related adverse events (AEs) were predominantly low grade and transient (Grade 1-2). The most frequently reported AEs included fatigue, low-grade fever, injection-site reactions, and flu-like symptoms. Importantly, vaccine-related immune activation seldom necessitated dose modification or treatment discontinuation (7, 9, 21, 22).

**Figure 3:**
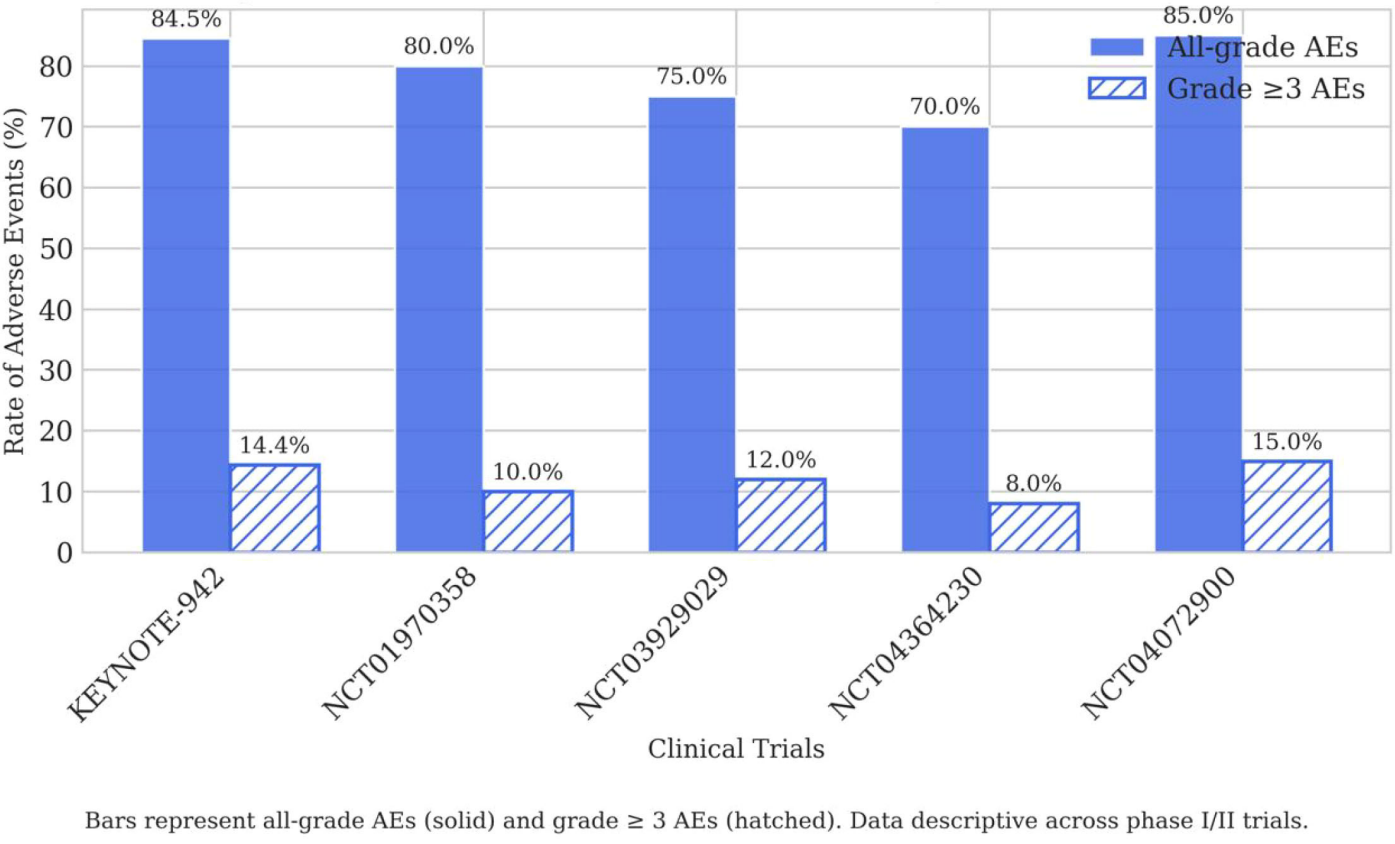
Adverse event profile across neoantigen vaccine platforms. X-axis, Clinical Trials (KEYNOTE-942, NCT01970358, NCT03929029, NCT04364230, NCT04072900); Y-axis, Rate of Adverse Events (%). Bars represent all-grade AEs (solid) and grade ≥3 AEs (hatched).

In melanoma studies such as KEYNOTE-942, the addition of the vaccine to pembrolizumab resulted in a serious (Grade ≥3) AE rate of 14.4%, which was found to be comparable to the pembrolizumab-alone arm (14.0%). This suggests that the high-grade toxicity primarily reflects the immune checkpoint inhibitor (ICI) co-administration rather than significant additive toxicity from the vaccine component itself. As shown in **Figure 3**, other key trials reported similar Grade ≥3 AE rates, generally ranging from 8% to 15% (9, 19).

Notably, no cytokine release syndrome or severe hypersensitivity events were documented across the reviewed trials, underscoring the favourable tolerability profile of personalised neoantigen vaccines (7, 9, 21).

However, with increasing use of combination regimens, particularly vaccines plus PD-1 blockade, additive or synergistic immune-related adverse events (irAEs) have emerged as a clinical concern. These include autoimmune thyroiditis, hepatitis, and dermatitis, largely manageable with corticosteroid tapering. Long-term safety data remain scarce, emphasizing the need for longitudinal pharmacovigilance registries and real-world monitoring beyond 24-month follow-up (4, 5, 9).

Overall, neoantigen vaccines demonstrate a favourable safety profile that allows for combination with ICIs without substantial overlapping toxicity, yet integration mandates vigilant management of irAEs and robust post-trial surveillance (9).

**Figure 4:**
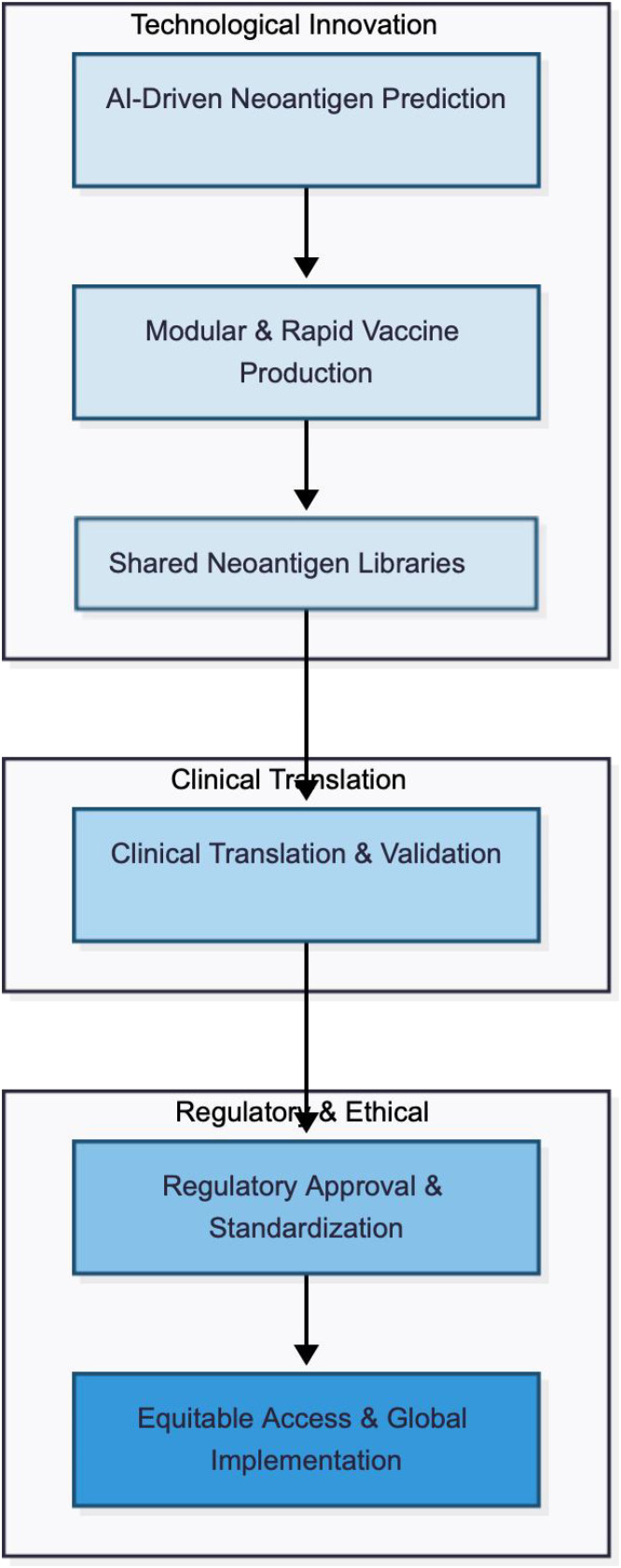
Future outlook for neoantigen vaccines. This diagram shows trends: AI prediction → modular production → shared antigen libraries, with arrows indicating development roadmap, including technological, clinical, and regulatory milestones.

**Figure 5:**
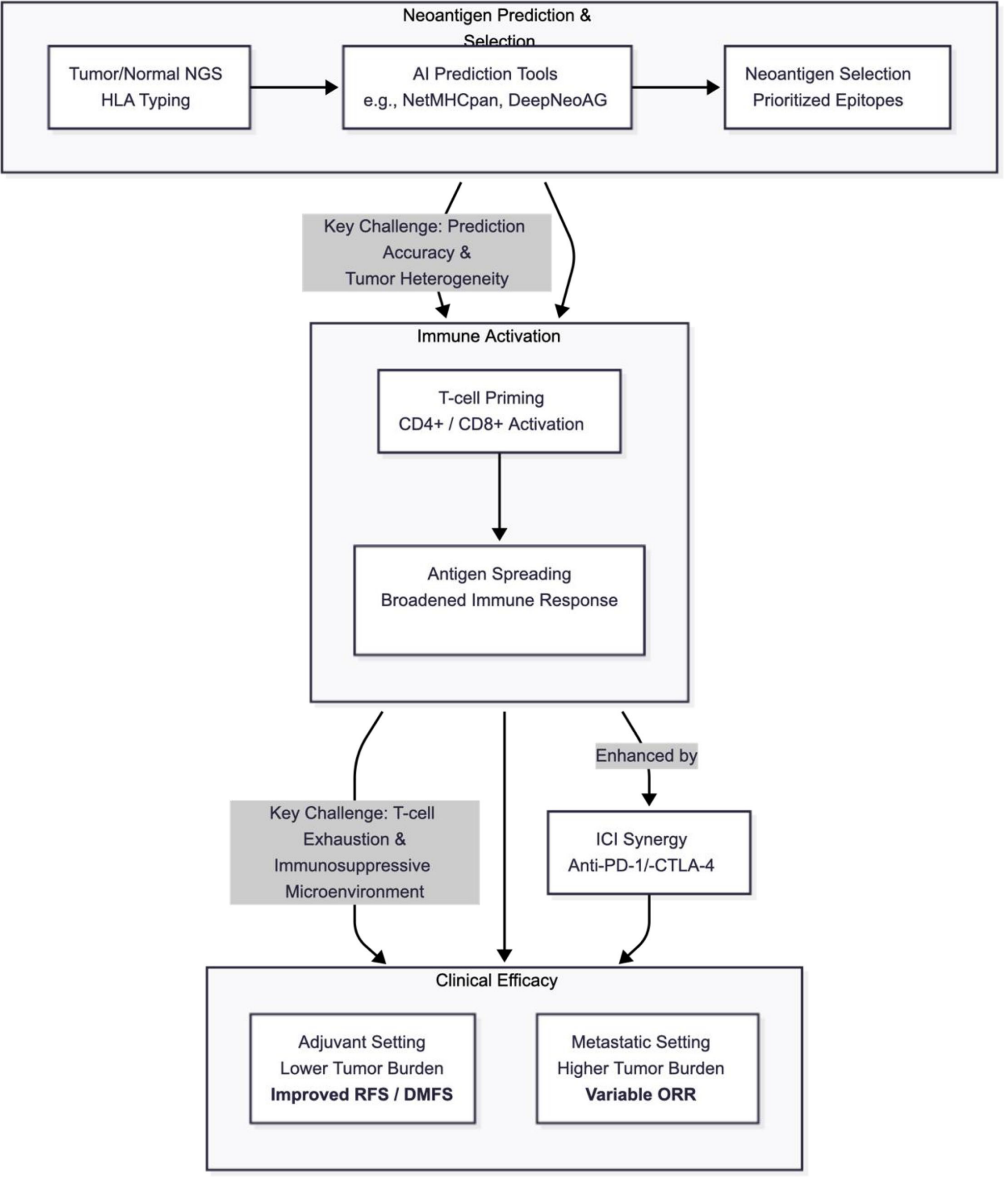
Mechanistic-clinical integration model. Box diagram linking neoantigen prediction (AI tools, MHC binding) → immune activation (T-cell priming, antigen spreading) → clinical efficacy (RFS/ORR improvements via ICI synergy). Arrows show causal flows; challenges (e.g., evolution) as barriers. This model illustrates how mechanistic strengths translate to adjuvant vs. metastatic differences.

**Figure 6:**
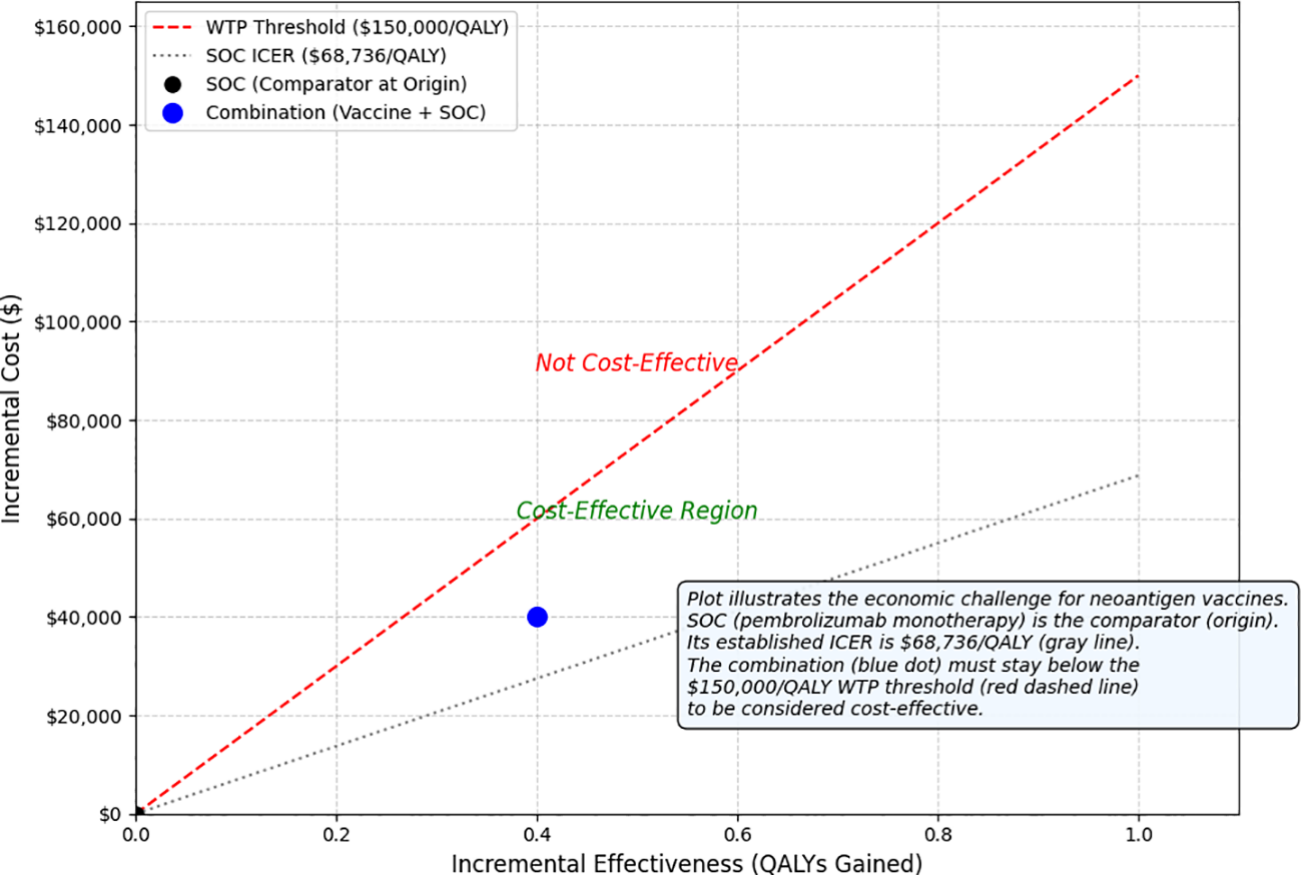
Cost-effectiveness plane for an add-on personalised neoantigen vaccine in the adjuvant setting. The origin represents pembrolizumab monotherapy (standard of care; comparator). The blue point illustrates the incremental cost and incremental QALYs of adding a personalised neoantigen vaccine to pembrolizumab, evaluated against pembrolizumab alone. The red dashed line denotes a willingness-to-pay (WTP) threshold of $150,000/QALY; points below the line are cost-effective under this threshold. The grey dotted line is shown for context only, reflecting a published U.S. estimate of pembrolizumab versus observation (ICER $68,736/QALY; resected stage IIB/IIC melanoma model) and should not be interpreted as the add-on comparator for the combination. Transferability of this benchmark to higher-risk resected populations depends on baseline recurrence risk, duration of benefit, and downstream treatment costs (41, 43).

### 6.1 Operational and value barriers to clinical adoption

#### 6.1.1 Vein-to-vein time and scalability

The dominant operational constraint for personalised neoantigen vaccines is the “vein-to-vein” interval, defined as the elapsed time from tumour sampling to first vaccine dose. Current real-world estimates of ~8–16 weeks are difficult to reconcile with oncology care pathways, particularly outside slow-moving adjuvant settings (16).

V940-001 (NCT05933577) illustrates the scalability problem at trial scale: a global Phase III program requiring individualised manufacturing for 1,089 patients has already experienced a multi-year timeline extension, consistent with throughput limits, cold-chain site constraints, and non-trivial batch attrition that can force re-manufacture (10).

The practical takeaway is that “clinical efficacy” is partly operational: even a potent vaccine can lose real-world effectiveness if delivery is routinely delayed beyond the window where recurrence risk is front-loaded or where metastatic dynamics outpace production.

#### 6.1.2 Clinical implications of the wait window

This manufacturing lag creates a clinically meaningful “wait window” in which patients may progress, relapse, or become ineligible for the therapy being produced for them.

It also imposes a psychological burden that is not incidental: prolonged uncertainty and perceived time-loss are repeatedly described as emotionally destabilizing in analogous personalised-cell-therapy workflows, and the same mechanism applies here (40).

#### 6.1.3 Cost-effectiveness constraints

Even if operational delivery is solved, reimbursement hinges on incremental value over an already expensive and effective standard of care. In the United States, published cost-effectiveness models in resected stage IIB/IIC melanoma estimate pembrolizumab (adjuvant) versus observation at approximately $68,736 per QALY gained (2022 USD; lifetime horizon) (41).

For a personalised vaccine added on top of pembrolizumab, the relevant decision question becomes the incremental cost-effectiveness of (vaccine + pembrolizumab) versus pembrolizumab alone under an explicit willingness-to-pay (WTP) threshold. ICER’s Reference Case commonly reports thresholds in the $100,000–$150,000 per QALY/evLY range (42).

This creates a value headroom (value-based price ceiling) problem: the maximum allowable incremental cost of adding the vaccine must remain below the WTP-constrained headroom (**Figure 6**). The headroom available specifically for vaccine pricing must also cover non-drug add-ons (tumour/normal sequencing, bioinformatics, bespoke manufacturing, cold chain).

Headroom methods are commonly used in early health-economic evaluation to quantify this price ceiling under explicit assumptions.

These estimates are based on resected stage IIB/IIC models and may not directly generalize to higher-risk resected populations without sensitivity analyses on baseline recurrence risk, duration of benefit, and downstream treatment costs.

Therefore, modest gains in recurrence-free survival (RFS) that translate into small QALY gains can fail payer thresholds unless (i) manufacturing and sequencing costs fall, and/or (ii) longer follow-up demonstrates durable survival gains that increase QALY benefit and downstream cost offsets (reduced relapse treatment costs) (41–43).

#### 6.1.4 Regulatory adaptation

Regulators are increasingly treating these products less like a conventional “one molecule, one label” drug and more like a controlled process: expedited designations exist, but the approval bottleneck shifts toward demonstrating that the AI-driven selection pipeline and individualised CMC controls are valid, auditable, and reproducible at scale (16–18, 44, 45).

## 7 Conclusion and future outlook

Personalised neoantigen vaccines have matured from a theoretical concept into a validated therapeutic class. The evidence from melanoma trials demonstrates consistent safety and robust immunogenicity, with clear signals of clinical efficacy, particularly in the adjuvant setting. The randomized KEYNOTE-942 trial (mRNA) and the durable responses in the AI-guided NCT05309421 trial (peptide) serve as powerful proofs-of-concept (9, 19, 28).

However, broad clinical adoption remains contingent on solving three interconnected bottlenecks. First, the prediction bottleneck persists, as the field still faces a reproducibility crisis and a validation gap between strong *in vitro* prioritization metrics and meaningful *in vivo* clinical benefit. Second, the biological bottleneck remains substantial, because even a well-designed vaccine may fail in immune-excluded or immunosuppressed tumours, or when vaccine-induced responses are blunted by concomitant medications such as dexamethasone. Third, the bench-to-bedside bottleneck continues to constrain translation, as current 8–16 week manufacturing timelines pose operational, clinical, and psychological burdens, while reimbursement will ultimately depend on whether incremental recurrence-free survival justifies the added cost (12, 16, 26, 32, 34, 37, 40).

Future progress will depend not only on better neoantigen selection algorithms, but also on rational therapeutic integration and scalable delivery. While vaccine-checkpoint inhibitor combinations currently represent the most clinically validated strategy in melanoma, emerging preclinical evidence suggests that radiotherapy, oncolytic virotherapy, and epigenetic modulation may further enhance antigen release, dendritic-cell activation, and T-cell trafficking (**Figure 4**). At the same time, the Phase III V940–001 trial will serve as the major translational inflection point for the field, testing whether the recurrence-free survival benefit observed in earlier studies can be preserved when individualised manufacturing is scaled beyond specialised clinical-trial settings. In parallel, regulatory evaluation is increasingly shifting toward validation of the process itself, including AI-assisted antigen selection and individualised CMC control (9, 10, 13, 16–18, 39, 44, 45).

Mechanistically, the current evidence argues against a rigid platform binary in which mRNA vaccines are equated with CD8^+^ immunity and peptide vaccines with CD4^+^ immunity. Instead, platform design, peptide length, adjuvant context, and cross-presentation efficiency collectively shape the balance between helper and cytotoxic T-cell responses. If ongoing advances in AI-guided prioritization, manufacturing scalability, and regulatory standardization can be achieved, personalised neoantigen vaccines may transition from experimental precision therapies to a durable component of oncology practice over the coming decade (10).

## 8 Limitations of this review

This review is a narrative synthesis and not a systematic review or meta-analysis. The search was limited to English-language publications and trial data available up to October 2025. The reliance on data from small, single-arm, and, in some cases, conference abstracts (e.g., NCT04364230, V940-001 3-year update) introduces a high risk of selection and reporting bias (**Table 4**), which may overstate treatment benefits. No primary data analysis was performed (10, 19, 22, 24).
