# Supplementary material for: Neoantigen-based cancer vaccines: a mechanistic and clinical review of personalised melanoma immunotherapy
Source: Front Immunol. 2026 Apr 2;17:1808146. doi: 10.3389/fimmu.2026.1808146 (PMC13083070; doi:10.3389/fimmu.2026.1808146)
Supplement: Supplementary file 1 [file Table1.docx]

Supplementary Material

# Supplementary Data

**Supplementary Table 1: Baseline and Outcome Data for Key Melanoma Neoantigen Vaccine Trials**

This table provides baseline characteristics and outcomes for the six key clinical trials reviewed in this article, contextualizing the relationship between vaccine platform, AI prediction sophistication, and clinical efficacy. Data correspond to Figure 2B in the main text.

| **Trial ID** | **Vaccine Platform** | **AI Integration Level** | **n** | **Disease Stage** | **Principal Endpoint** | **Efficacy Outcome** | **Baseline TMB** |
| --- | --- | --- | --- | --- | --- | --- | --- |
| KEYNOTE-942 (NCT03897881) | mRNA (mRNA-4157) + Pembrolizumab | High (Deep Learning) | 157 | Adjuvant (Resected Stage IIIB–IV) | RFS | RFS HR 0.510 (95% CI 0.288–0.906); DMFS HR 0.384 | High (Melanoma) |
| NCT05309421 (EVX-01) | Peptide (EVX-01) + Pembrolizumab | High (Deep Learning) | 16 | Metastatic (Unresectable) | ORR | 75% ORR (12/16); 92% (11/12) sustained at 24 mos. | High (Melanoma) |
| NCT01970358 (NeoVax) | Peptide (NeoVax) + Poly-ICLC | Low (Rule-based) | 8 | Adjuvant (Resected Stage IIIB–IV) | RFS (Disease-Free) | 75% disease-free (6/8) at ~4 yrs | High (Melanoma) |
| NCT03929029 | Peptide (NeoVax) + Montanide + Ipi/Nivo | Low (Rule-based) | 11 | Metastatic | ORR | 36% ORR (4/11); responses in 8/11 | High (Melanoma) |
| NCT04072900 | Peptide + Toripalimab (Anti-PD-1) | Low (Rule-based) | 30 | Metastatic | ORR | 10% ORR (3/30) | High (Melanoma) |
| NCT04364230 | Peptide (Shared Ag + neoAg-mBRAF) + Adjuvants | Low (Rule-based) | 22 | Adjuvant (Resected, Disease-Free) | Immunogenicity | No efficacy endpoint; 27% CD4+ response to shared Ag; poor neoantigen immunog. | High (Melanoma) |

**Notes:**

AI Integration Level: Inferred from published descriptions. “High (Deep Learning)” denotes trials explicitly using deep-learning–guided neoantigen prediction pipelines; “Low (Rule-based)” denotes trials using conventional binding-affinity algorithms (e.g., NetMHCpan).

KEYNOTE-942 (mRNA-4157): Classified as ‘High (Deep Learning)’ based on the AI-guided mRNA vaccine design platform.

NCT05309421 (EVX-01): Explicitly described as using deep-learning–guided neoantigen selection, with reported 81% accuracy in predicting T-cell responses.

NCT01970358 & NCT03929029 (NeoVax): Early peptide trials (circa 2017) using conventional rule-based affinity predictors.

NCT04072900 & NCT04364230: Classified as ‘Low (Rule-based)’ given their use of conventional prediction methods.

Baseline TMB: Melanoma is generally a high-TMB tumour type; cross-trial differences in baseline TMB may be less pronounced, but direct comparability is limited by inconsistent reporting across studies.
